# Primary choriocarcinoma of the renal pelvis presenting as intracerebral hemorrhage: a case report and review of the literature

**DOI:** 10.1186/1752-1947-5-501

**Published:** 2011-10-05

**Authors:** Fani Kyriakou, Michael M Vaslamatzis, Styliani Bastani, Maria Alexandra Lianou, Christina Vourlakou, Antonia Koutsoukou

**Affiliations:** 1 1st Critical Care Department and Pulmonary Services, Evangelismos General Hospital, University of Athens Medical School, Ipsilantou 45-47, 10676 Athens, Greece; 2 Oncology Department, Evangelismos General Hospital, Ipsilantou 45-47, 10676 Athens, Greece; 3 Pathology Department, Evangelismos General Hospital, Ipsilantou 45-47, 10676 Athens, Greece

## Abstract

**Introduction:**

A choriocarcinoma is a malignant neoplasm normally arising in the gestational trophoblast, gonads and, less frequently, the retroperitoneum, mediastinum and pineal gland. Primary choriocarcinomas of the renal pelvis are extremely rare.

**Case presentation:**

We report a case of primary choriocarcinoma of the renal pelvis in a 38-year-old Greek woman of reproductive age, presenting with a sudden development of intracerebral hemorrhage due to metastatic lesions. The diagnosis was established with a renal biopsy, along with an elevated serum level of beta-human chorionic gonadotropin. An extensive diagnostic work up confirmed the origin of the choriocarcinoma to be the renal pelvis.

**Conclusion:**

Extragonadal choriocarcinomas are rare neoplasms that require extensive laboratory and imaging studies to exclude a gonadal origin. Moreover, this is the first case of severe intracerebral hemorrhage as the initial presentation of primary choriocarcinoma of the renal pelvis. Nonetheless, choriocarcinomas should be considered in the differential diagnosis of women of reproductive age.

## Introduction

A choriocarcinoma is a malignant neoplasm normally arising in the gestational trophoblast and gonads. Choriocarcinomas of the renal pelvis are extremely rare and they often present in association with transitional cell carcinoma (TCC). Their presenting symptoms are flank pain and macrohematuria. We present a case of primary choriocarcinoma of the renal pelvis in a woman of reproductive age presenting with sudden development of intracerebral hemorrhage.

## Case presentation

A 38-year-old Greek woman was transferred to the Emergency Department of our hospital in a comatose state. After intubation, an urgent computed tomography (CT) scan of her brain was performed, which revealed a 7.8 × 3.3 × 5.0 cm intracerebral hematoma located at her left occipitoparietal area with surrounding edema and a midline shift.

Our patient had a history of two pregnancies that were normal term Cesarean deliveries, the first four years ago and the second 10 months ago. The rest of her medical history was unremarkable.

An emergent craniotomy was performed along with evacuation of the hematoma. After surgery, she was transferred to the Intensive Care Unit (ICU). Our patient remained sedated and mechanically ventilated for neuroprotection, while an intraparenchymal monitoring device was inserted for continuous monitoring of her intracranial pressure. Intracranial hypertension was treated with osmotic diuretics, hyperventilation and hypertensive therapy, aiming to maintain a constant cerebral perfusion pressure higher than 60 mmHg.

A laboratory workup, including liver and renal function tests, coagulation screening, fibrinogen and full blood count, were normal. Due to the enlargement of the right hilus found on a chest X-ray, extensive CT imaging studies were performed, which revealed a space occupying lesion at the inferior pole of her right kidney (approximately 3 cm), a solitary nodule at the IVa part of her liver consistent with a metastatic lesion, a soft tissue mass situated in the right hilus of her lung enhanced by intravenous contrast and diffusely distributed chest nodules without intrathoracic lymph nodes.

Her serum level of β-chorionic gonadotropin (β-hCG) was over 200,000 mIU/mL and this value was repeatedly confirmed. Nonetheless, a pelvic examination was unremarkable and all the imaging studies (pelvic ultrasonography, including transvaginal ultrasonography and pelvic CT) revealed the normal appearance of her uterus and bilateral ovaries.

During the next two weeks, our patient demonstrated gradual improvement of her general status and she was able to open her eyes spontaneously.

Magnetic resonance imaging of her brain showed, apart from postsurgical lesions at the area of the hematoma, a metastatic lesion situated on her left temporal lobe, whereas a magnetic angiography did not reveal any vascular dysplasia.

On the 26^th ^day of her ICU stay, our patient underwent an ultrasound guided fine needle aspiration of the hepatic lesion in order to establish a histological diagnosis for possible further treatment. Due to consequent intra-abdominal bleeding, she was given surgical radiofrequency ablation of the hepatic lesion. A right nephrectomy was also performed due to the size of the lesion with evidence of imminent bleeding.

On the 35^th ^day she had a new episode of massive intracerebral hemorrhage, confirmed by CT, which was considered inoperable by our neurosurgeons. She died 10 days later, due to septic shock.

**Figure 1:**
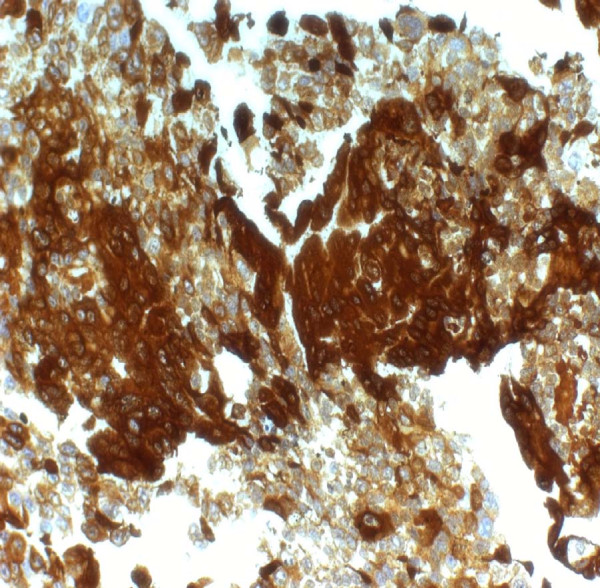
**Right kidney (renal pelvis-pelvicalyceal system)**. Intense (+++) immunohistochemical staining for β-HCG (magnification × 400).

**Figure 2:**
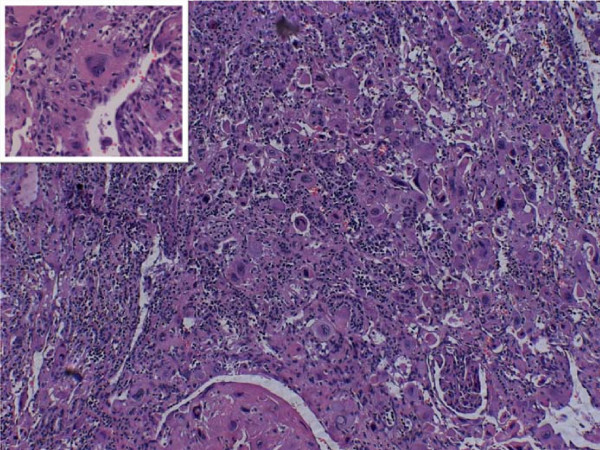
**Right kidney (renal pelvis-pelvicalyceal system)**. Intense presence of syncytiotrophoblasts and trophoblasts in a carcinomatous area (hematoxylin and eosin stain; magnification × 200).

Macroscopic examination following the right nephrectomy revealed the presence of a grey-red tan encapsulated tumor, which measured 6 × 3.5 × 3 cm, situated at the inferior pole, invading the renal pelvis-pelvicalyceal system and extending to the renal capsule. The histological examination of this mass showed the presence of a malignant neoplasm composed of syncytiotrophoblastic and cytotrophoblastic cells in an extensively hemorrhagic and necrotic background with subsequent cystic degeneration and numerous neoplasmatic vascular emboli (Figures 1 and 2). Immunohistochemical staining revealed intense expression of β-hCG (Figure 1) and placental alkaline phosphatase, although it was negative for epithelial membrane antigen (EMA) and thyroid transcription factor-1 (TTF-1) expression. All above findings are indicative of a choriocarcinoma of the renal pelvis. The hepatic biopsy revealed neoplastic invasion of the liver by a tumor histologically identical to the choriocarcinoma of her renal pelvis.

## Discussion

Choriocarcinoma is a rare malignant tumor of genital cells that develops usually in the uterus, the ovaries or the placenta of women after molar or, rarely, after normal gestation and in the testis of men. Choriocarcinomas disseminate rapidly through the hematogenous route; the lungs, brain and liver are the distant organs more often involved [1].

Extragonadal choriocarcinomas are scarce neoplasms. They have been described in isolated cases, located in the gastrointestinal system, the lungs, the urinary bladder and the pineal gland [2,3].

Primary choriocarcinoma of the renal pelvis, as described in this case report, is extremely rare. Slightly more frequent, although rare too, are choriocarcinomas in association with high grade TCC. The cases described in the international literature are very few and their characteristics are highlighted in Table 1.

**Table 1:** Summary of the characteristics of reported cases of choriocarcinoma.

Case	Reference	Age	Sex	Material	Histologic type	Serum HCG	Tissue HCG	Outcome (months)
**1**	[4]	54	M	Kidney	TCC-CC	ND	++	A6
**2**	[5]	56	F	Kidney	CC	ND	NA	A22
**3**	[6]		M	Kidney	CC	NA	NA	D4
**4**	[2]	84	M	Kidney	TCC-CC	ND	NA	NA
**5**	[3]	68	M	Kidney	TCC-CC	High	NA	NA
**6**	[7]	60	M	Kidney	TCC-CC	ND	NA	D1,5
**7**	Present	38	F	Kidney-Liver	CC	High	+++	D1

A: alive; CC: choriocarcinoma; D: dead; F: female; M: male; NA: not available; ND: not done; TCC: urothelial transitional cell carcinoma.

Campo *et al. *detected the presence of hCG-immunoreactive cells in nine (19%) out of 47 patients with high grade urothelial carcinoma. In only one of those cases was the neoplasm situated in the renal pelvis and the 54-year-old patient had been treated with nephrectomy. Histological examination of this tumor revealed a transitional cell carcinoma with choriocarcinomatous elements [4]. Grammatico *et al. *described the case of a TCC with choriocarcinomatous differentiation involving the pelvoureteric junction of the kidney in an 84-year-old man with hematuria [2]. Vahlensieck *et al*. reported β-hCG-positive extragonadal germ cell neoplasia of the renal pelvis in a 56-year-old woman with a history of flank pain on the right side for three years [5]. Huang *et al*. described the case of a choriocarcinoma of the right kidney in a man presenting with fever, gross hematuria and flank pain [6]. In all above mentioned cases, preoperative serum levels of β-hCG are not available, while it is not mentioned if there were metastases. Hara *et al. *presented the case of a 68-year-old man with hematuria diagnosed with a high grade TCC with choriocarcinomatous component of his left renal pelvis [3]. More recently, Zettl *et al. *reported the case of a tumor of the renal pelvis composed of papillary urothelial carcinoma in association with choriocarcinoma, in a 60-year-old male patient presenting with painless macrohematuria. Chromosomal analysis revealed a close genetic relationship between these two carcinomatous components. This led the authors to suggest that the choriocarcinoma may have resulted from clonal evolution of the urothelial carcinoma. As in our case, widespread hepatic and pulmonary metastases were found in this patient, while the β-hCG levels in his serum were elevated [7].

According to the predominant theories, extragonadal choriocarcinomas arise from multipotent cells left behind during early embryologic development, from dedifferentiation of neoplastic urothelial transitional cells, or from a metaplastic procedure [2].

Pathologically, the tumors have been described as large and exophytic, often showing hemorrhage and necrosis. In all cases, the presence of choriocarcinoma was indicated by the presence of syncytiotrophoblastic elements. In accordance with previous studies, we observed giant syncytiotrophoblasts with intense β-hCG and placental alkaline phosphatase positive reactivity, while extensive necrosis was a predominant feature.

The differential diagnosis between choriocarcinomas of the urothelial system and mixed choriocarcinomas associated with TCC, on histological grounds, is very difficult and it is based on strong expression of β-hCG. Moreover, EMA is positive for TCC but negative for choriocarcinoma [8].

Extensive clinical and laboratory research is needed in order for the primary extragonadal localization to be confirmed [5]. In the present case, the histological and immunohistochemical characteristics, along with the location of the disease, the absence of tumor in the gonads, the negativity of TTF-1 expression by the tumor cells and the absence of intrathoracic lymph nodes [9] were absolutely compatible with a *de novo *choriocarcinoma of the renal pelvis.

Interestingly, in our case, there was a marked absence of symptoms from the urothelial system, especially hematuria, which is in contrast with the international literature [3]. Severe intracerebral hemorrhage as an initial presentation of primary choriocarcinoma of the urothelial system has not been described so far. Intracerebral hemorrhage represents the commonest clinical appearance of primary choriocarcinomas of the genital system with cerebral metastasis [10]. The trophoblastic carcinomas are supplied by fragile vessels and simultaneously have the tendency to invade and destroy the wall of these vessels, thus provoking bleeding. Cerebral metastases are associated with a worse prognosis.

Since all choriocarcinomas contain and produce hCG, serum β-hCG values have been used for the diagnosis and follow up of extragonadal localizations (breast, lung, kidney and stomach). Serum values of β-hCG in our patient were repeatedly over 200,000 mIU/mL.

Extragonadal choriocarcinomas are usually diagnosed when they have metastasized. The surgical excision proposed by the literature is performed either for localized disease or for establishing the final diagnosis [3,5,7]. In our case, the presenting symptoms, the extent of the disease, the bad performance status and co-existent infection prohibited further treatment.

## Conclusions

We present the case of a 38-year-old woman with primary choriocarcinoma of the renal pelvis and severe intracerebral hemorrhage. To the best of our knowledge, this is the first case of extragonadal choriocarcinoma described that presented as an intracerebral hemorrhage. Clinicians should thus be conscious of this atypical clinical presentation in women of reproductive age.

## Abbreviations

β-hCG: beta-chorionic gonadotropin; CT: computed tomography; EMA: epithelial membrane antigen; ICU: intensive care unit; TCC: transitional cell carcinoma; TTF-1: thyroid transcription factor-1.

## Consent

Written informed consent was obtained from the patient's next-of-kin for the publication of this case report and any accompanying images. A copy of the written consent is available for review by the Editor-in-Chief of this journal.

## Competing interests

The authors declare that they have no competing interests.

## Authors' contributions

FK participated in all medical interventions, collected the data and drafted the manuscript. MMV drafted the final version of the manuscript. SB participated in all medical interventions and drafted the final version of the manuscript. MAL participated in the diagnosis. CV participated in the diagnosis. AK participated in all medical interventions and drafted the final version of the manuscript. All authors read and approved the final manuscript.
